# Commentary: Titi semantics: Context and meaning in Titi monkey call sequences

**DOI:** 10.3389/fpsyg.2019.00512

**Published:** 2019-03-14

**Authors:** Swan Commier, Mélissa Berthet

**Affiliations:** Institut Jean Nicod, Département d'études Cognitives, ENS, EHESS, CNRS, PSL Research University, Paris, France

**Keywords:** semantics, formal linguistics, titi monkey, vocalizations, primate, alarm

Analyses of non-human vocal systems inspired by formal linguistics have recently emerged and appear fruitful to help understand the semantic properties of animal vocalizations (Schlenker et al., 2016b). The vocal system of one primate species has been particularly illuminated by such a perspective, the Black-fronted titi monkeys (*Callicebus nigrifrons*). This species uses two alarm calls, the A-call and the B-call, and arranges them into diverse sequences. The composition of those sequences differs depending on the type and location of the predator (Cäsar et al., 2013), as shown in (1):

(1) a. A raptor in a non-ground position (flying or perched in the canopy) elicits a sequence of A-calls, (hereafter A+)
b. A raptor on the ground elicits a sequence of A-calls followed by B-calls (A+B+)
c. A cat in the canopy elicits a sequence a single A-call followed by B-calls (AB+)
d. A cat on the ground /the presence of a terrestrial animal/ a monkey descending near the ground elicits a sequence of B-calls only (B+).

Schlenker et al. (2017) proposed that (i) an A-call refers to a serious non-ground threats; (ii) a B-call is given to noteworthy events; (iii) an Informativity Principle mandates that the more informative A-call should be used instead of the less informative B-calls whenever it applies to the situation—a principle independently motivated in similar investigations in other species, such as Campbell's monkeys and Putty-nosed monkeys (Schlenker et al., 2016b). Moreover, Schlenker et al. (2017) stated that each call that is produced constitutes an independent utterance and reflects the state of the environment at the exact time of production. In that sense, a combination of calls is viewed as a sequence of calls that each have their independent meaning, regardless of their position and the other calls composing the sequence. Thus, Schlenker et al. (2017) derived the following analysis (2) for the generalizations in (1):

(2) a. A+ is given to a serious non-ground threat
b. a raptor on the ground attacks by flying, hence it is treated as a non-ground threat (A+) that stops being serious at some point because it is not in a hunting position (hence B+)
c. a cat in the canopy is a serious non-ground threat (hence A). However, the cat's hunting strategy relies on surprise, and spotting a felid may deter it from attacking (Zuberbühler et al., 1999). Thus, after the emission of one A-call, the cat stops being a serious threat for it has been spotted and may stop hunting: A-calls cannot be used anymore, and B+ is therefore uttered
d. in ground and/or non-serious alerts only B can be used.

However, Cäsar (2011) argued that aerial predators represent the major danger to Titi monkeys, which suggests that the meaning “serious non-ground threat” of A-calls is redundant. We argue that the specification of the location can be eliminated from the meaning of A-call, resulting in a simplification such as A-calls refer to “serious threat.” The generalizations in (1) can thus be analyzed as follow (3):

(3) a. A+ is given to a serious threat
b. A raptor on the ground is still a dangerous threat (A+) but stops being serious at some point for it is not in a hunting position (hence B+)
c. a cat in the canopy is a serious threat (A) that stops being serious once spotted (B+)
d. B are used in non-serious alerts.

In Schlenker et al. (2017), A-calls referred to the level of alarm (“serious”) *and* the location of the threat (“non-ground”) while we argue that A-calls only refer to the level of alarm. Our analysis is therefore more parsimonious, and in line with the animal communication literature that described several animal communication systems as urgency-based (e.g., Rauber and Manser, 2017).

When hearing A-calls, titi monkeys mainly look upwards (Cäsar et al., 2012). Schlenker et al. (2017) suggested that the semantics of A-calls comprised a “non-ground” component that could explain the behavioral reaction of the monkeys. However, we think that environmental knowledge actually provides a better explanation to account for the observed behavior: if serious threats usually come from above and titi monkeys know this, then the most adaptive reaction to hearing a conspecific signaling “serious alert” is to look upwards.

We reckon that decisive field experiments are now necessary to verify the meaning of A-calls. One could, for instance, present titi monkeys that are on the ground with a cat model. According to Schlenker et al. (2017), this would elicit B-calls, because the cat is not a serious non-ground threat when on the ground. Our revised theory predicts that it would elicit A-calls, because the cat on the ground becomes a serious threat when the monkeys are not in the trees.

Beyond reflecting on the titi monkeys alarm system, our own analysis proves that the approach introduced by Schlenker et al. (2016a,b) and encouraged elsewhere (e.g., Seyfarth and Cheney, 2016) is likely to be very productive in the future. Their approach allows the building of clear hypotheses, that can be criticized and revised, and that can lead to very clear predictions on the semantics of animal systems that can eventually be tested in the field.

## Author Contributions

SC: conception and first draft; MB: further redaction and revision of the manuscript.

### Conflict of Interest Statement

The authors declare that the research was conducted in the absence of any commercial or financial relationships that could be construed as a potential conflict of interest.
